# Challenging fungal infections in cystic fibrosis: a case of mixed Aspergillus species infection and antifungal combination testing

**DOI:** 10.1099/acmi.0.000758.v3

**Published:** 2024-04-19

**Authors:** Laís Pontes, Franqueline Reichert-Lima, Ana Luisa Perini Leme Giordano, Maria Luiza Moretti, Angélica Zaninelli Schreiber

**Affiliations:** 1 School of Medical Sciences - University of Campinas, Campinas, São Paulo, Brazil; 2 School of Medical Sciences in São José dos Campos – Humanitas, São José dos Campos – São Paulo, Brazil

**Keywords:** *Aspergillus*spp, Cystic fibrosis, azole antifungals, antifungal combination

## Abstract

*Aspergillus* stands as the predominant fungal genus in the airways of cystic fibrosis (CF) patients, significantly contributing to their morbidity and mortality. *Aspergillus fumigatus* represents the primary causative species for infections, though the emergence of rare species within the *Aspergillus* section *Fumigati* has become noteworthy. Among these, *Aspergillus lentulus* is particularly significant due to its frequent misidentification and intrinsic resistance to azole antifungal agents. In the management of invasive aspergillosis and resistant infections, combination antifungal therapy has proven to be an effective approach. This report documents a case involving the death of a CF patient due to a pulmonary exacerbation linked to the colonization of multiple *Aspergillus* species, including *A. lentulus*, *A. fumigatus*, and *A. terreus*, and treated with Itraconazole (ITC) monotherapy. We delineated the procedures used to characterize the *Aspergillus* isolates in clinical settings and simulated *in vitro* the impact of the combination antifungal therapy on the isolates obtained from the patient. We evaluated three different combinations: Amphotericin B (AMB)+Voriconazole (VRC), AMB+Anidulafungin (AND), and VRC+AND. Notably, all strains isolated from the patient exhibited a significant decrease in their minimum inhibitory concentration (MIC) or minimum effective concentration (MEC) values when treated with all antifungal combinations. The VRC+AMB combination demonstrated the most synergistic effects. This case report emphasizes the critical importance of susceptibility testing and precise identification of *Aspergillus* species to enhance patient prognosis. It also underscores the potential benefits of combined antifungal treatment, which, in this case, could have led to a more favourable patient outcome.

## Data Summary

The accession numbers of the strains are LIF 2352 – PP262156, LIF 2486 - PP266452, LIF 2552 – PP266453 and, LIF 3104 – PP26645. All raw data that were generated in this work are available in the manuscript itself.

## Introduction

Cystic fibrosis (CF) is the most common autosomal hereditary disease, causing high morbidity and mortality in the Caucasian population. It is caused by mutations in the transmembrane conductance regulator (CFTR) gene, affecting the functionality of multiple organs. Specifically, dysfunction of the CFTR gene in lung epithelial cells leads to dysregulation of electrolyte flow and the accumulation of hyperviscous mucus. Consequently, this increases the risk of bacterial and fungal respiratory infection, rendering individuals susceptible to chronic infections and triggering exacerbated inflammatory responses [1,2].

*Aspergillus fumigatus* is the most prevalent mould in the respiratory tracts of CF patients and several studies have shown a significant increase in resistance to triazole antifungals, which are the first-line drugs for treating aspergillosis [3]. *A. fumigatus* contains allergens that stimulate antigen-presenting cells and T lymphocytes within the adaptive immune system, leading to hypersensitivity diseases, including sinusitis, bronchitis and allergic bronchopulmonary aspergillosis (ABPA) [4]. Recently, the International Society of Human and Animal Mycology (ISHAM) has recognized that CF is a predisposing condition of ABPA [5].

Patients with CF receive broad-spectrum antibacterial therapies through oral, intravenous, and daily nebulizer treatments, both for prophylaxis and the treatment of respiratory symptoms. Previous studies have reported a connection between nebulizer treatments using antibacterial drugs and *Aspergillus* isolation from the respiratory tract [6,7].

*Aspergillus fumigatus* belongs to the *Aspergillus* section *Fumigati*, which comprises several species, including the cryptic species * A. lentulus* [8]. These two species cannot be distinguished based on their similar micromorphology; discrimination is only possible by molecular methods. *A. lentulus* exhibits reduced susceptibility to the commonly used antifungal agents and is frequently resistant to Voriconazole (VRC) [9].

Studies have demonstrated the superior efficacy of antifungal combination therapy over monotherapy for the management of fungal infections [10,13]. This study is focused on reporting a case involving the death of a CF patient due to an acute pulmonary exacerbation associated with a multispecies *Aspergillus* infection, which includes *A. lentulus*. Our objectives include detailing the characterization procedures used for the *Aspergillus* isolates in the clinical setting and conducting *in vitro* testing to assess the impact of combination antifungal therapy on all isolates retrieved from the patient.

## Case presentation

In this report, we document the case of a woman in her early twenties diagnosed with cystic fibrosis at 11 months of age due to a high sweat chloride level (103-129 meq l^−1^), followed by the identification of a homozygous CFTR mutation (ΔF508/ΔF508).

**Fig. 1.**
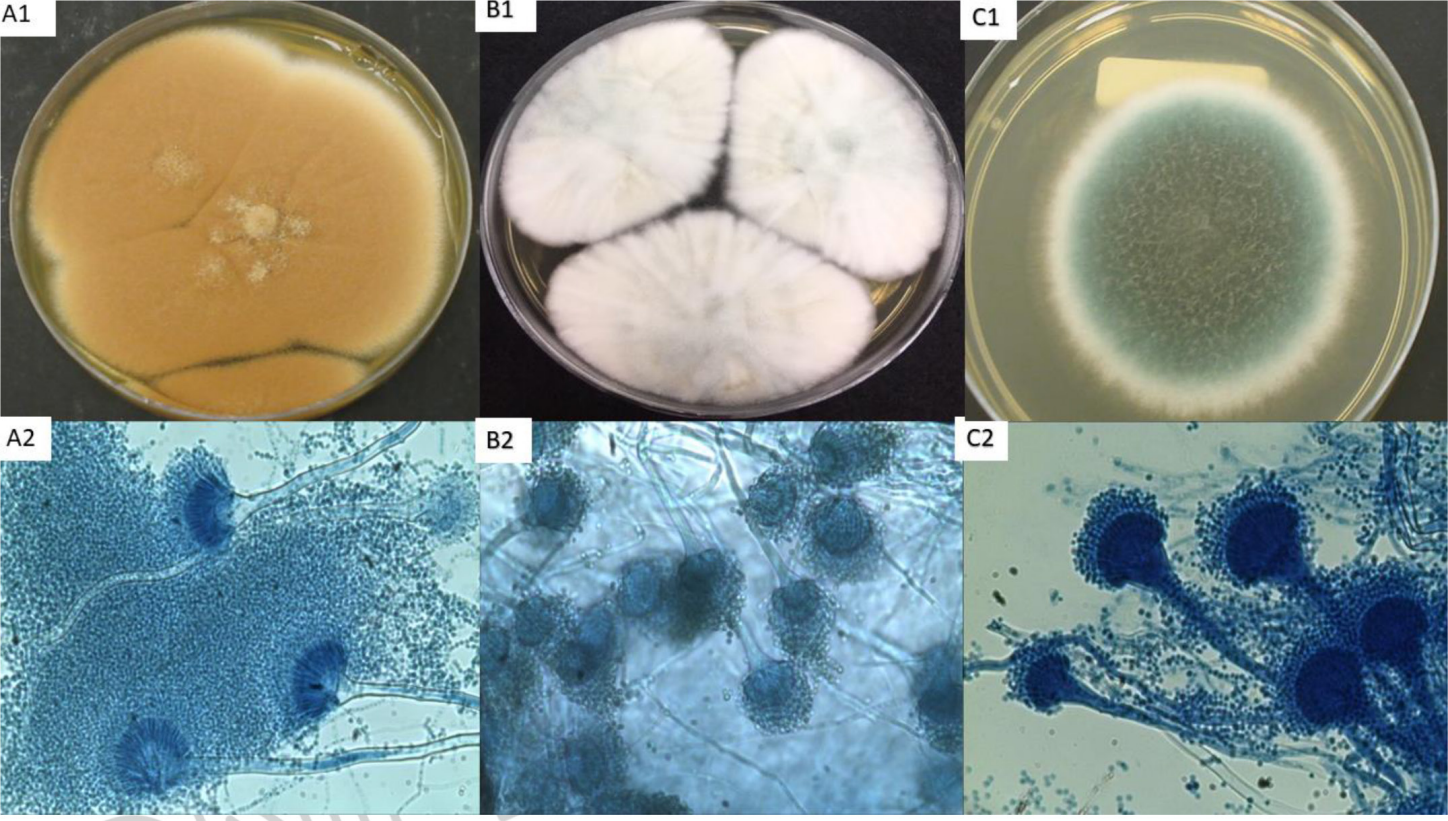
*Aspergillus* spp. isolated from sputum samples in Agar Sabouraud Dextrose. A1 and A2 *A. terreus*: colony after 48 h at 37 °C and microscopic aspect with blue lactophenol staining. B1 and B2 *A. lentulus*: colony after 7 days at 37 °C and microscopic aspect with blue lactophenol staining. C1 and C2 *A. fumigatus*: colony after 48 h at 37 °C and microscopic aspect with blue lactophenol staining.

Up to the point of presentation, the patient had not experienced any lung infections associated with bacteria or fungi. However, in the same year, her health deteriorated, and she developed pancreatic insufficiency and worsening pulmonary symptoms. The first isolation of *Aspergillus* sp. was from a sputum sample, revealing the presence of *A. terreus* (LIF 2486) (Fig. 1) and *P. aeruguniosa. A. terreus* was considered colonization, and treatment with Ciprofloxacin 500 mg per day for 15 days was initiated, along with Tobramycin 3 mg per day. The patient’s clinical condition significantly improved, leading to her discharge after intravenous antibiotic treatment.

However, 3 months after the first isolation of *Aspergillus* sp. the patient’s respiratory condition worsened, leading to another hospital admission. Sputum analysis identified *P. aeruginosa* and two *Aspergillus* species resembling *A. fumigatus*. One of them was confirmed *A. fumigatus* (LIF 2554) based on macro and micromorphology characteristics. The second isolate exhibited distinctive features, after 10 days of incubation at 37 °C, including a whitish cotton colony with abundant septate and hyaline hyphae, along with a few conidiophores with uniseriate vesicles. This isolate was sent to sequence the β-tubulin 2A/B genes, confirming it as the cryptic species *A. lentulus* (LIF 2354) (Fig. 1).

Due to clinical worsening, the patient was diagnosed with ABPA based on clinical characteristics and elevated IgE levels. Treatment was initiated with Ciprofloxacin 500 mg per day and Itraconazole 200 mg twice a day for 15 days. Following clinical improvement, the patient was discharged and continued oral Itraconazole 200 mg per day at home for 15 days.

In the following years*, P. aeruginosa* colonization continued and there were no more *Aspergilus* sp. isolates until 3 years later. Due to *P. aeruginosa* presence, this leads to a progressive decline in lung capacity and necessitates the initiation of oxygen therapy. The patient used Tobramycin 3 mg per day continuously during this period.

After 3 years, the patient was readmitted to the emergency room due to worsening respiratory symptoms, and respiratory failure was diagnosed. Sputum analysis revealed the presence of *A. fumigatus* (LIF 3104) by sequencing β-tubulin 2A/B genes, which was isolated on the day of admission. The patient’s condition rapidly deteriorated, leading to a diagnosis of acute pulmonary exacerbation caused by *A. fumigatus* and ultimately resulting in the patient’s death (Fig. 1).

**Fig. 2.**
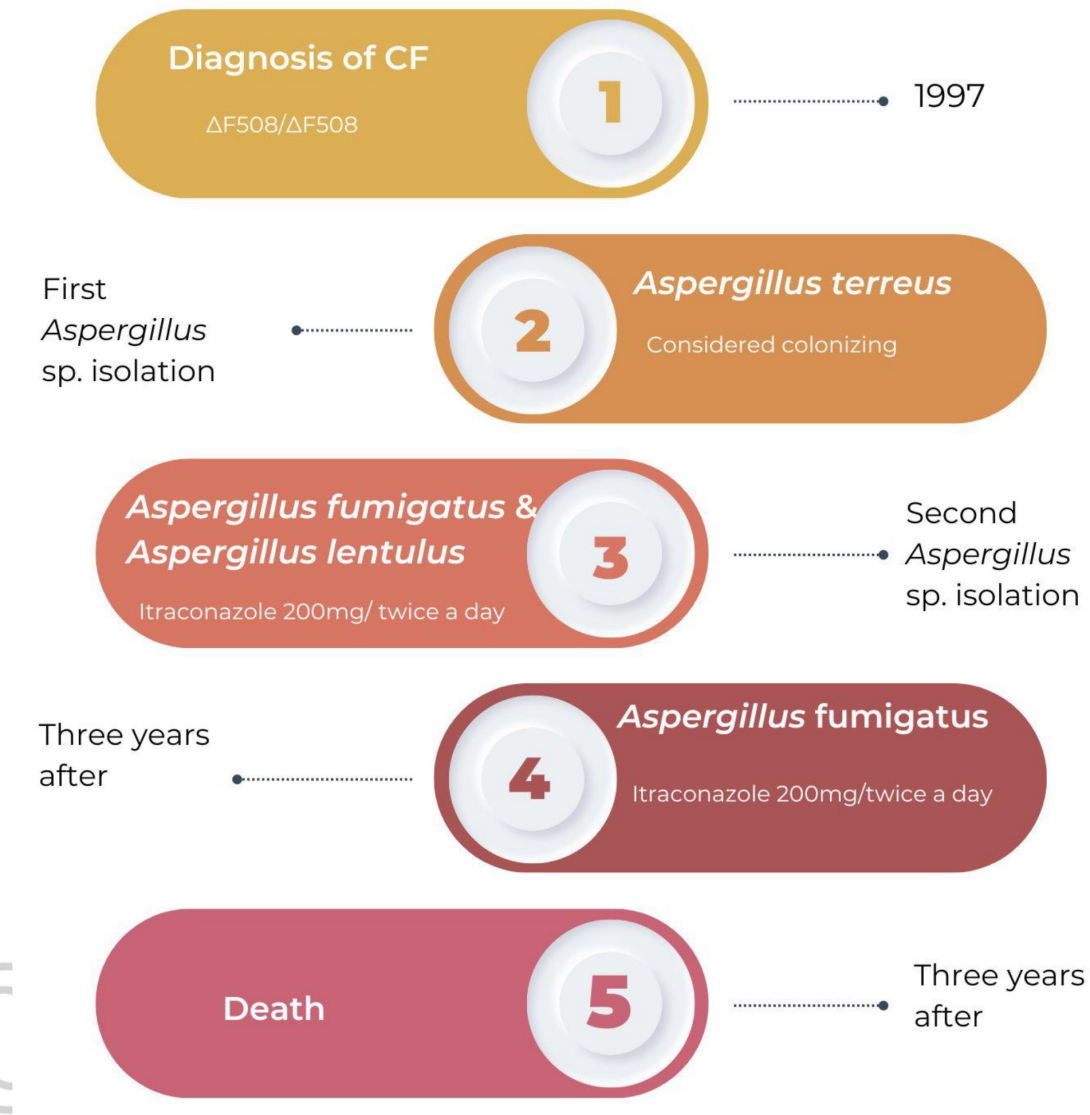
Timeline of *Aspergillus* species infections.

In Fig. 2 it’s possible to observe the timeline of the case description.

Susceptibility testing was conducted using the broth microdilution assay following the Clinical and Laboratory Standards Institute (CLSI) M38-A2 guidelines [14]. The MICs for individual antifungal drugs are listed in Table 1.

**Table 1.** Minimum inhibitory concentration and minimal effective concentration (µg ml^−1^) of six antifungal agents tested against *Aspergillus* species

LIF	Species	MIC/MEC (µg ml^−1^)					
		MCF	CPF	AMB	ITC	VRC	POS
2486	*A. terreus*	≤0.015	0.25	2	0.5	1	0.5
2354	*A. lentulus*	≤0.015	0.25	2	0.5	8	0.25
2554	*A. fumigatus*	≤0.015	0.25	1	0.5	1	0.5
3104	*A. fumigatus*	≤0.015	0.25	2	1	1	0.5

AMB Amphotericin B CPF caspofungin ITC itraconazole MCF micafungin MEC Minimal effective concentration MIC Minimum inhibitory concentration POS posaconazole VRC voriconazole

We evaluated combinations of antifungal drugs *in vitro* to study their interactions. We undertook combination testing in adherence to international guidelines, which prescribe this approach in instances of therapeutic failure. The tests were performed using the checkerboard broth microdilution method. Three different drug combinations were assessed: Amphotericin B (AMB)+Voriconazole (VRC), AMB+Anidulafungin (AND), and VRC+AND. To prepare the drug solutions, AMB, VRC, and AND (Sigma‐Aldrich) were dissolved as per the manufacturer’s instructions and diluted in RPMI 1640 (Sigma‐Aldrich). Drug dilutions ranged from 0.06 to 8 µg ml^−1^ for AMB and VRC, and 0.0075 to 1 µg ml^−1^ for AND. Plates were incubated at 37 °C for 24 h and 48 h. They were quantitatively evaluated using the fractional inhibitory concentration (FiC) index, calculated as follows: FiC = (MIC A in combination/MIC A) + (MIC B in combination/MIC B). Interaction outcomes were defined as synergistic if FiC ≤0.5, indifferent if FiC >0.5 to ≤4, and antagonistic if FiC index exceeded 4.0 as commonly used in previous studies [15] (Table 2).

**Table 2.** Fractional inhibitory concentration (FiC) results for all the isolates and evaluated

LIF	Species	FiC results					
		VRC+AND	Interaction	VRC+AMB	Interaction	AND+AMB	Interaction
2486	*A.terreus*	1.25	I	0.09	S	1.5	I
2354	*A lentulus*	0.07	S	0.15	S	0.31	S
2554	*A.fumigatus*	0.37	S	0.12	S	0.75	I
3104	*A.fumigatus*	0.56	I	0.53	I	0.56	I

AMB Amphotericin B AND Anidulafungin FiC Fractional Inhibitory Concentration I Indifferent S Synergy VRC Voriconazole

We observed varying effects of drug combinations on different *Aspergillus* isolates:

1. The combination of VRC and AMB demonstrated a synergistic effect on three isolates, namely LIF 2354 (*A. lentulus*), LIF 2486 (*A. terreus*), and LIF 2554 (*A. fumigatus*), while it had an indifferent effect on LIF 3104 (*A. fumigatus*).
2. The interaction between VRC and AND showed a synergistic effect on LIF 2354 (*A. lentulus*) and LIF 2554 (*A. fumigatus*) and an indifferent effect on LIF 2486 (*A. terreus*) and LIF 3104 (*A. fumigatus*).
3. Combining AND and AMB yielded a synergic effect on LIF 2354 (*A. lentulus*) and had an indifferent effect on LIF 2486 (*A. terreus*), LIF 2554 (*A. fumigatus*) and LIF 3104 (*A. fumigatus*).

It is noteworthy that the MIC values decreased for all isolates when the drugs were combined. Importantly, all drug combinations assessed resulted in a synergic effect for *A. lentulus*. In this study, all *in vitro* interactions were effective, and no antagonism was detected.

In Table 3 it’s possible to observe all concentrations of MIC and MEC values of each drug, both alone and in combination.

**Table 3.** Minimum inhibitory concentration (MIC) and Minimal effective concentration (MEC) in µg ml^−1^ of antifungal agents alone and combinations against clinical *Aspergillus* sp. isolates

LIF	Species	VRC+AND MIC/MEC range (µg ml^−1^)			
		MIC VRC	MIC VRC comb	MEC AND	MEC AND comb
2486	*A. terreus*	1	0.25	0.015	0.015
2354	*A. lentulus*	8	0.06	0.125	0.0075
2554	*A. fumigatus*	1	0.25	0.06	0.0075
3104	*A. fumigatus*	1	0.5	0.125	0.0075
**LIF**	**Species**	**VRC+AMB MIC range (µg ml^−1^**)			
		**MIC VRC**	**MIC VRC comb**	**MIC AMB**	**MIC AMB comb**
2486	*A. terreus*	1	0.06	2	0.06
2354	*A. lentulus*	8	1	2	0.06
2554	*A. fumigatus*	1	0.06	1	0.06
3104	*A. fumigatus*	1	0.5	2	0.06
**LIF**	**Species**	**AMB+AND MIC/MEC range (µg ml^−1^**)			
		**MIC AMB**	**MIC AMB comb**	**MEC AND**	**MEC AND comb**
2486	*A. terreus*	2	1	0.015	0.015
2354	*A. lentulus*	2	0.5	0.125	0.0075
2554	*A. fumigatus*	1	0.25	0.06	0.03
3104	*A. fumigatus*	2	1	0.125	0.0075

AMB Amphotericin B AND anidulafungin comb minimal inhibitory concentration in combination comb Minimal effective concentration in combination MEC Minimal effective concentration MIC minimal inhibitory concentration VRC voriconazole

## Discussion

*Aspergillus*, particularly *A. fumigatus*, is the most commonly found fungal genus found in the airways of CF patients. Additionally, *P. aeruginosa* is frequently isolated from these patients and is a major cause of morbidity and mortality. Notably, recent findings have suggested that *A. fumigatus* may also contribute to the poor prognosis of CF lung disease [16].

Regarding treatment recommendations, the Infectious Diseases Society of America (IDSA) 2016 guidelines recommend VRC as the first-line treatment for invasive aspergillosis. Liposomal AMB and Isavuconazole are considered alternative options. Echinocandins can be used in salvage therapy, either alone or in combination, but they are not recommended as monotherapy for the primary treatment of invasive aspergillosis. It’s worth mentioning that while the IDSA does not recommend primary therapy with echinocandin, the European Society of Clinical Microbiology and Infectious Diseases (ESCMID) occasionally recommends it as the primary treatment for this condition [17].

Antifungal combination therapy is often employed for refractory aspergillosis, the antifungal combination therapy may be used as primary or salvage therapy to enhance the treatment outcomes, whether as a primary or salvage strategy. Commonly used combinations include VRC or AMB paired with an echinocandin. This choice is rooted in the potential for synergistic interactions, as these drug classes have distinct mechanisms of action: VRC and AMB affect cell membrane function, while echinocandins affect cell wall function [17].

In the scenario, we conducted a study to explore whether the use of antifungal agents could potentially reduce the MIC and VRC susceptibility of *A. lentulus*. To our surprise, the most synergistic effect was observed when combining VRC and AMB.

In our study, combination tests produced notably lower MIC and MEC values *in vitro* for most drugs compared to the drugs alone, even in cases with indifferent interactions. Given the frequent failures associated with monotherapy for *Aspergillus* infections [17], our results suggest that antifungal combination therapy could be a valuable treatment strategy.

Supporting this notion, a randomized, double-blind multicenter trial involving 454 patients with haematological malignancies and suspected or documented invasive aspergillosis showed that primary treatment with VRC and AND or led to lower mortality rates (13.3 %) compared to VRC monotherapy (27.5 %) within 6 weeks [18].

For *A. lentulus*, the MIC of VRC was 8 µg ml^−1^ when tested alone. However, when VRC was combined with AMB and AND the MIC dropped to 1 µg ml^−1^. Three isolates, including *A. lentulus*, *A. terreus*, and one *A. fumigatus*, had a MIC of 2 µg ml^−1^ for AMB tested individually, but when combined, the MICs reduced to 0.06 µg ml^−1^ for all isolates (as shown in Table 3). These results indicate that the combination of these drugs requires lower and less toxic concentrations to inhibit microorganisms, which is advantageous for the infection outcomes.

The findings align with existing literature. Infections caused by *A. fumigatus* are known to be more lethal and fatal than those caused by other *Aspergillus* species, even cryptic species within its Section. Factors such as the production of secondary metabolites, virulence, and the development of resistance and persistence *in vivo* may have contributed to the patient’s death [19,20].

The presence of *Aspergillus* spp. in CF patients demands careful attention, as *A. fumigatus* is linked to exacerbated inflammatory responses and decreased lung function. Additionally, cryptic species with reduced susceptibility can lead to ABPA and the common practice of monotherapy may prove ineffective. In this context, the role of the antifungal combination therapy should be considered.

Our study reveals that combining drugs results in lower inhibitory concentrations for microorganisms, suggesting the potential efficiency of combination therapy for complex infections and resistant species in CF patients. However, further clinical trials and studies are necessary to explore antifungal combinations and gather more data on this approach.

## Abbreviations

ABPA: allergic bronchopulmonary aspergillosis
A. fumigatus: Aspergillus fumigatus
A. lentulus: Aspergillus lentulus
AMB: Amphotericin B
AND: Anidulafungin
A. terreus: Aspergillus terreus
CF: Cystic Fibrosis
CFTR: transmembrane conductance regulator
CPF: Caspofungin
ITC: Itraconazole
MCF: Micafungin
MEC: Minimal effective concentration
MIC: Minimium Inibitory Concentration
P. aeruginosa: Pseudomonas aeruginosa
POS: Posaconazole
VRC: Voriconazole
